# Vitamin E (α-Tocopherol): Emerging Clinical Role and Adverse Risks of Supplementation in Adults

**DOI:** 10.7759/cureus.78679

**Published:** 2025-02-07

**Authors:** Alan D Kaye, Austin S Thomassen, Sydney A Mashaw, Ellie M MacDonald, Aubert Waguespack, Lily Hickey, Anushka Singh, Deniz Gungor, Anusha Kallurkar, Adam M Kaye, Sahar Shekoohi, Giustino Varrassi

**Affiliations:** 1 Department of Anesthesiology, Louisiana State University Health Sciences Center, Shreveport, USA; 2 School of Medicine, Louisiana State University Health Sciences Center, Shreveport, USA; 3 Department of Pharmacy Practice, Thomas J. Long School of Pharmacy and Health Sciences, University of the Pacific, Stockton, USA; 4 Department of Pain Medicine, Fondazione Paolo Procacci, Rome, ITA

**Keywords:** antioxidant, cancer, cardiovascular, hemorrhage, stroke, vitamin e, α-tocopherol

## Abstract

Vitamin E, primarily in its active form α-tocopherol, is a well-known antioxidant that protects cells from oxidative stress and free radical damage. It plays an essential role in maintaining cellular integrity and supporting immune function, making Vitamin E a widely popular and easily accessible dietary supplement for overall health and wellness. However, high-dose Vitamin E supplementation has become a concern related to potential risks. The scientific research surrounding the safety and efficacy of Vitamin E is complex yet emphasizes a balance in the use of Vitamin E supplementation. Excessive or high-dose supplementation causes a shift in this balance, as Vitamin E's beneficial antioxidant properties are outweighed by harmful interference in normal cellular processes such as immunity, cell growth, and oxidative stress. An additional complication involves Vitamin E's anticoagulant effects, which have been shown to amplify the risk of bleeding when high-dose supplementation is combined with blood thinners such as warfarin and aspirin. Studies have linked high-dose Vitamin E supplementation to adverse outcomes, including enhanced risks of all-cause mortality, hemorrhagic stroke, cardiovascular events, and certain cancers. These risks are particularly significant for individuals with pre-existing health conditions such as heart failure, coagulation disorders, or a history of stroke. The potential risk of adverse side effects emphasizes the need for further research into high-dose Vitamin E supplementation. This review will provide a comprehensive analysis of Vitamin E's multifaceted role in health and physiology, focusing on navigating the balance between potential benefits and risks in supplementation.

## Introduction and background

Vitamin E, a fat-soluble vitamin and antioxidant, has long been celebrated for its role in promoting health and preventing disease. Found abundantly in nuts, seeds, and green leafy vegetables, this essential nutrient has multiple forms, with α-tocopherol being the most biologically active. As a crucial component of the human diet, Vitamin E plays a pivotal role in maintaining cellular integrity and immune system function, with its antioxidant properties offering protection against oxidative damage caused by free radicals [1]. Despite its established importance, Vitamin E's use in the form of supplements has remained a topic of ongoing debate. Vitamin E is often highlighted as one of the key antioxidants in human nutrition, functioning as a protective shield against oxidative stress. Free radicals, unstable atoms produced during normal physiologic processes and in response to stress, can damage cellular DNA. This damage has been implicated in aging, chronic inflammation, and disease development. Vitamin E neutralizes these free radicals, preserving cellular health and contributing to the body's resilience against environmental and physiological stressors [2].

The recommended dietary allowance (RDA) for Vitamin E varies by age, gender, and physiological conditions. As of 2016, the RDA for most adults, according to the Food and Drug Administration (FDA), is 15 mg/day. This recommendation ensures sufficient antioxidant protection and supports the nutrient's secondary roles, such as enhancing immune function and promoting skin health [3]. Rich dietary sources of Vitamin E include almonds, sunflower seeds, peanuts, spinach, and avocados [4]. However, despite its availability in everyday foods, many individuals use Vitamin E supplements to meet their perceived health needs, especially when dietary habits are suboptimal. Vitamin E supplementation is commonly associated with various potential benefits, extending beyond essential nutritional roles. Contributions to immune function are well-documented, with the nutrient helping modulate immune responses, especially in older adults whose immune function can decline [5]. Vitamin E's ability to support skin health has also made it a popular ingredient in dietary supplements and topical formulas [6]. Finally, antioxidant properties have fueled widespread belief that supplementation can help to prevent or slow the progression of chronic diseases such as heart disease, cancer, and neurodegenerative diseases [7]. While these claims have widely popularized Vitamin E supplementation, scientific evidence supporting them remains complex and contradictory.

The public perception of Vitamin E has been overwhelmingly positive, driven by its natural occurrence in nutrient-rich foods and its image as a safe, effective antioxidant. Vitamin E supplements are widely marketed as general wellness enhancers, often appealing to consumers seeking to bolster health and proactively prevent disease. Unlike prescription medications, dietary supplements are usually perceived as less risky and more accessible. Vitamin E's ubiquitous presence in multivitamins has also reinforced this perception, making it a common choice for individuals aiming to address multiple health concerns in one fell swoop [3]. Today, Vitamin E's popularity spans demographics, appealing to young and old alike, as well as to athletes and individuals with specific health conditions [8,9]. Despite its widespread use, the actual health benefits of Vitamin E supplementation are now being questioned as more rigorous scientific investigations have failed to confirm many of the early claims regarding efficacy [8].

## Review

Controversy surrounding benefits and risks

While Vitamin E has long been regarded as a cornerstone of antioxidant therapy, its status has been scrutinized recently due to conflicting evidence regarding health benefits and potential risks. Early observational studies, which often linked higher Vitamin E intake to reduced chronic disease risks, were largely anecdotal and based on associations rather than causation [10,11]. These studies laid the foundation for Vitamin E's popularity but did not account for many confounding factors that could influence health outcomes.

Subsequent randomized controlled trials (RCTs) and meta-analyses have painted a more complex picture, often failing to replicate earlier findings. For example, while initial studies highlighted the use of Vitamin E in the prevention of cardiovascular disease, more recent trials show no correlation between high levels of Vitamin E and cardiovascular disease prevention or the prevention of mortality related to cardiovascular disease [12]. In some cases, high-dose Vitamin E supplementation has even been associated with adverse effects, such as an enhanced risk of bleeding and hemorrhagic stroke related to Vitamin E's anti-vitamin K effects, as well as an enhanced risk of bladder cancer [13,14]. These findings have sparked a reevaluation of Vitamin E supplementation, prompting researchers and healthcare professionals to reconsider its role in preventative medicine. The evolving evidence underscores the need for a more nuanced understanding of how Vitamin E interacts with the body, particularly in hypervitaminosis [15].

Vitamin E is not alone in facing heightened scrutiny: other antioxidants, such as vitamin C, have also been reexamined for their purported benefits [16]. This growing skepticism reflects a broader shift in the scientific community's approach to antioxidant therapy, emphasizing the importance of evidence-based practices over theoretical claims. The dichotomy between Vitamin E's well-established nutritional importance and the uncertain benefit of supplementation highlights the need for ongoing research and public education. Consumers should be informed about the potential risks of high-dose supplementation while remaining clear on the contexts in which Vitamin E supplementation may still offer value. For example, individuals with specific deficiencies or medical conditions, such as Alzheimer's, may benefit from targeted supplementation [17]. However, for the general population, the emphasis should likely shift toward achieving adequate Vitamin E intake through a balanced diet rich in whole foods [3].

Vitamin E's popularity as a supplement reflects its enduring fascination with the promise of preventative medicine, even as science continues to refine our understanding of its true impact [18]. By exploring the intricate balance between benefits and risks, the present investigation aims to provide a comprehensive overview of Vitamin E's role in human health. This review will explain the mechanisms of harm of Vitamin E supplementation, including its interference with blood clotting and interactions with other vitamins, its potential cause of cardiovascular disease and hemorrhagic stroke, and its potential links to cancer.

Mechanism of harm from Vitamin E supplementation

The Antioxidant Paradox

The 'antioxidant paradox' has been used to describe the observations that oxygen radicals and reactive oxygen species (ROS) contribute to several diseases; however, large doses of antioxidants, such as Vitamin E, have proved to have little or no preventative or therapeutic effects. A balance of free radicals and antioxidants is essential for proper cellular function. If free radicals increase out of proportion, damage to lipids, proteins, and DNA could occur and cause several diseases. Conversely, high amounts of antioxidants have been shown to affect health negatively [19]. One of the theories for this is that cells normally exist in a reduced state but require some oxidation for essential functions. Many gene transcription factors require intermittent oxidation to stimulate cell proliferation [20]. Apoptosis, another crucial cellular function, is stimulated by oxidation, but paradoxically, too much oxidation will inactivate caspase enzymes and inhibit apoptosis [21]. Transition metals are a byproduct of oxidative damage from metalloproteins and can initiate free radical damage, especially in the reduced state. Antioxidants, such as Vitamin E, given after transition metals have already created oxidative damage, could cause more damage by reducing them [21].

ROS are secondary mechanisms for intracellular signaling cascades that mediate cell growth, autophagy, and inflammatory and immune functions. In response to invading pathogens, phagocytic neutrophils and macrophages produce ROS for eradication [22]. Oxidative stress causes the p53-regulated cyclin-dependent kinase inhibitor, p21, to become activated, causing cells to arrest in the G0, which halts cell division [23]. ROS produced from radiation can also cause activation of the tumor-suppressor retinoblastoma protein (Rb), stopping cell cycle progression [24]. Excessive Vitamin E supplementation can eliminate the ROS that activates these cell survival pathways, potentially leading to increased cellular proliferation and the inability to eliminate infectious pathogens.

Early studies provided evidence that high-dose Vitamin E supplementation increased plasma oxidation by up to 27% [25]. A landmark 2005 meta-analysis found a relationship correlating dosage and all-cause mortality, especially increased risks with dosages greater than 150 IU/day, and stated that high-dosage (> or = 400 IU/day) Vitamin E supplements should be avoided due to the increased risk of all-cause mortality [26]. Proposed mechanisms of Vitamin E that are attributed to increasing all-cause mortality are the pro-oxidant effect on low-density lipoproteins that can create α-tocopheroxyl radicals, displacing fat-soluble antioxidants leading to the disruption of the balance between antioxidants and oxidative stress, and inhibiting cytosolic glutathione S-transferases that help with drug and endogenous toxin elimination [27-29].

Recently, evidence has been published suggesting that Vitamin E can cause DNA damage and promote cell transformation frequency [30]. The Selenium and Vitamin E Cancer Prevention Trial (SELECT) showed a 17% increase in prostate cancer incidence in the Vitamin E arm compared to the placebo [31]. Premalignant prostate epithelial organoids studied have supported the findings in SELECT that Vitamin E promoted cell proliferation and survival, promoting tumorigenesis in the early stages of cancer [32]. Another mechanism of Vitamin E contributing to the increased risk of prostate cancer is the activation of cytochrome p450 enzymes, making prostate cells more vulnerable to pro-mutagenic and pro-carcinogenic agents [30].

Interference With Blood Clotting Mechanisms

Vitamin E also exhibits anticoagulative properties by reducing platelet aggregation and interfering with vitamin K-dependent clotting factors. Vitamin E inhibits protein kinase C (PKC), reduces platelet cyclooxygenase activity, and inhibits lipid peroxidase formation [33,34]. In addition, Vitamin E quinone, the main oxidation product of Vitamin E, inhibits vitamin K-dependent carboxylase required to activate vitamin K-dependent clotting factors, which are essential in the blood clotting cascade [35].

In this regard, patients on blood-thinning medications, in addition to high serum Vitamin E levels, potentially from high-dose supplementation, are at a higher risk of bleeding than patients on blood-thinning medications alone. In a study of people on the anticoagulant warfarin, the investigators found a Vitamin E serum level-dependent increase in bleeding. The study showed that there was an increased risk of minor bleeding with 5.16±1.91 μmol/mmol cholesterol, P=0.006, and major bleeding with 5.72±2.0 μmol/mmol cholesterol, P=0.008 [36]. High-dose Vitamin E supplementation is therefore contraindicated in people on anticoagulants.

A 2010 study suggested there was an increased risk of hemorrhagic stroke with the use of Vitamin E supplementation by 22% and reduced risk of ischemic stroke by 10% [37]. The results of this study caution against unnecessary Vitamin E supplementation, given the larger risk and relatively small benefit in strokes. Conversely, a 2020 meta-analysis stated insufficient evidence to conclude that Vitamin E increases hemorrhagic stroke risk [37]. Additionally, a 2024 meta-analysis supports the finding that Vitamin E's role in stroke prevention is inconclusive [38]. Therefore, there is a need for more studies before a conclusion about the correlation between Vitamin E and hemorrhagic stroke can be made.

Interaction With Other Fat-Soluble Vitamins

High-dose Vitamin E supplementation over 1000 mg/day (1500 IU/day of the natural form or 1100 IU/day of the synthetic form) can cause major bleeding events, including the potential for intracranial hemorrhage [15]. High-dose Vitamin E supplementation has also been known to cause decreased absorption of other fat-soluble vitamins, impairing bone mineralization, coagulopathies, and fat-soluble vitamin absorption [39]. Excessive Vitamin E can be detrimental to bone health by interfering with vitamin K metabolism and excretion, competitive binding, and blocking entry for the Vitamin E transporter protein with other Vitamin E isomers that are beneficial to the bone and prooxidant effects [40]. A study conducted at Harvard University found that rats given 'mega-doses' of Vitamin E developed 20% weaker bones than rats on a normal diet [41]. However, these effects have not yet been studied in humans. Coagulopathies, through the aforementioned mechanism in the previous section, occur by interfering with vitamin K. Vitamin E competes for the enzyme vitamin K epoxide reductase, which converts vitamin K to the active form. Therefore, vitamin K stays inactive, preventing gamma-carboxylation on vitamin K-dependent coagulation factors, inhibiting the clotting cascade, and increasing bleeding risk [42]. Additionally, Vitamin E decreases factor IX and platelet aggregation, further increasing the risk of coagulopathies. Lastly, fat-soluble vitamins are all absorbed in the small intestines. Thus, it is reasonable to conclude that high-dose Vitamin E supplementation could compete for absorption with other fat-soluble vitamins, leading to malabsorption or deficiency of vitamins A, D, and K.

Immune System Modulation

Among the other roles of Vitamin E in the body, it also plays a significant role in immune function. A recent meta-analysis suggested reduced C-reactive protein, and IL-6 was associated with α-tocopherol supplementation. However, it was noted that doses over 1000 mg/day were not effective in reducing subclinical inflammation and were not recommended [43]. At optimal dosage, Vitamin E immunoregulation is targeted at improving T-cell-mediated functions. Additionally, it enhances lymphocyte proliferation, IL-2 production, helper T-cell activity, natural killer cell activity, and macrophage phagocytotic activity. It also has indirect suppressive effects by downregulating prostaglandin E2, tumor necrosis factor-a, and IL-6 in response to pathogens [44]. It could be concluded that high doses of Vitamin E further suppress the immune response, causing immune dysfunction and an inability to fight off infections or diseases effectively. Several early studies have found that Vitamin E supplementation depresses the bactericidal activity of leukocytes and the mitogen-induced lymphocyte transformation [45]. This is theorized to be due to decreased levels of oxidative stress metabolites available to kill pathogens after being phagocytosed by polymorphonuclear leukocytes (PMNs) related to the antioxidant effects of Vitamin E [46]. High-dose Vitamin E, which creates an excessive antioxidant effect, could contradict the immune system by increasing T-cell modulation. Still, paradoxically, it could deplete ROS and intermediates needed to fight infectious pathogens.

Risks associated with high-dose Vitamin E supplementation

Total Mortality Risk

A 2005 meta-analysis that included 135,967 participants in 19 clinical trials, nine of which tested Vitamin E alone and the other 10 tested Vitamin E combined with other vitamins or minerals, showed results of increased all-cause mortality risk with varying Vitamin E dosages. A group of participants was given high-dosage supplementation of Vitamin E (400 IU/day), while another group was given low-dosage supplementation (<400 IU/day). The pooled all-cause mortality risk difference in participants tested with high-dosage Vitamin E supplementation resulted in 39 per 10,000 persons (95% confidence interval (CI), 3 to 74 per 10,000 persons; P=0.035). The risk difference in the participants tested with low-dosage Vitamin E supplementation was -16 per 10,000 persons (CI, -41 to 10 per 10,000 persons; P>0.2). After a dose-response analysis, it was determined that there was an enhanced risk of all-cause mortality associated with Vitamin E supplementation of dosages greater than 150 IU/day [26]. A possible reason for Vitamin E affecting mortality could be that, at high dosages, Vitamin E can displace other fat-soluble antioxidants [28]. The disruption of the normal antioxidant could make cells more vulnerable to oxidative damage [27]. Tolerable upper intake levels (UL) of Vitamin E (α‐tocopherol), based on data from the European Food Safety Authority (EFSA) of the general European population, have been updated, and it has been determined that the tolerable upper intake level for Vitamin E is 300 mg/day in healthy adults [47,48]. A 1999-2000 National Health and Nutrition Survey had participants answer questions about their intake of vitamins, minerals, and other dietary supplements; 11.3% of the 4609 adults who participated said they consumed at least 400 IU of Vitamin E daily. The survey showed that this intake increased with age, was more common in white Americans versus African Americans or Mexican Americans, and was about equal for men and women [49].

Adverse Cardiovascular Effects

In studies examining adverse cardiovascular events in patients taking high-dose Vitamin E, the HOPE and HOPE-TOO trials have shown an increase in heart failure rates among patients with vascular disease or diabetes mellitus. The HOPE trial randomized 9541 participants to Vitamin E or placebo over 4.5 years, and the HOPE-TOO trial was an extension trial with 7040 patients over 2.6 years. A regression model, which considered all HOPE study patients, identified treatment assignment to Vitamin E as an independent predictor for heart failure. A regression model that included all HOPE participants found that treatment with Vitamin E predicted heart failure (hazard ratio=1.13; CI=1.01-1.26; P=0.04). A sub-study that included 506 HOPE patients observed a mean (SD) decrease in left ventricular ejection fraction (LVEF) of 1.86% in the Vitamin E group and 0.58% in the placebo group. The mechanism by which Vitamin E contributes to a decrease in LVEF is unclear, but one possible explanation could be the potential for Vitamin E to disrupt myocardial function by becoming a pro-oxidant while in an oxidative environment [50]. Vitamin E has been seen to have an even larger effect on the risk of heart failure in cases with a history of myocardial infarction. The GISSI-Prevenzione trials studied the effects of Vitamin E on 8415 patients who had experienced myocardial infarction in the prior three months. In total, 4202 of the 8415 participants were treated with Vitamin E, while the rest were in the control group. After 3.5 years of observation, 220 participants had developed heart failure. The Vitamin E-assigned group showed a nonsignificant 20% increased risk of developing heart failure; however, participants in the Vitamin E-assigned group with poor ventricular function (LVEF <50%) had a significant 50% increased risk of heart failure. This finding supports the idea that Vitamin E can be even more dangerous if given as supplementation in patients with prior adverse cardiovascular events [51].

Risk of Stroke (Hemorrhagic vs. Ischemic)

A 2010 systematic review and meta-analysis of randomized, placebo-controlled trials investigated Vitamin E's effect on stroke incidents. Nine trials were included, totaling 118,765 participants, and concluded that Vitamin E enhanced the risk of hemorrhagic stroke by 22% and decreased the risk of ischemic stroke by 10% but had no effect on total stroke risk. The absolute risk is equivalent to one additional hemorrhagic stroke for every 1250 individuals taking Vitamin E and one ischemic stroke prevented per 476 individuals taking Vitamin E. The meta-analysis showed reduced ischemic stroke risk related to Vitamin E; however, the increase in hemorrhagic stroke risk with Vitamin E supplementation outweighs the reduction [52]. In a retrospective, observational study of patients with non-valvular atrial fibrillation (NVAF) receiving anticoagulant therapy (OAT), a population of 566 participants was analyzed using their serum cholesterol-adjusted Vitamin E (vit E/chol) to observe a possible relationship with risk bleeding events. The participants were treated with warfarin and serum Vitamin E. The study showed higher vit E/chol levels in participants who experienced bleeding than those who did not (5.27±1.93 vs. 4.48±1.97 μmol/cholesterol; P<0.001). This study shows that in patients with NVAF who receive OAT, Vitamin E serum levels can be used as a possible predictor of bleeding events and further supports the idea that Vitamin E has anticoagulant effects [53]. Vitamin E has been shown to disrupt vitamin K-dependent clotting factor activation, and it can stop the conversion of factor X to Xa by inhibiting the expression of tissue factors. Both aspects of Vitamin E could precipitate increased bleeding. Another way in which Vitamin E has been shown to affect bleeding is with antiplatelet properties. A previous study demonstrated that Vitamin E can inhibit platelet aggregation using an oxidative stress-mediated mechanism [35,36,54,55].

Potential Links to Cancer

There has been an increased risk of prostate cancer in men taking Vitamin E supplements. The SELECT trial studied the increase in risk for 35,533 men 50 years or older (African Americans) or 55 years or older (all others) with a prostate-specific antigen (PSA) ≤4.0 ng/mL and a digital rectal examination not suspicious for prostate cancer. The participants were given Vitamin E, selenium, or both, or a placebo. The trial observed an increased risk of prostate cancer for the Vitamin E-only assigned group (hazard ratio=1.17; 99% CI=1.004-1.36; P=0.008), supporting the conclusion that Vitamin E supplementation increases the risk of prostate cancer in healthy men [56]. In previous studies, high-dosage Vitamin E has shown the ability to suppress apoptosis through various mechanisms related to its ability to reduce oxidative stress. A study performed on rabbits suggested that high-dosage Vitamin E, when administered jointly with vitamin C, diminished oxidative stress, reduced caspase 9 and caspase 3 activities, and myocyte apoptosis. Through these mechanisms, high-dosage Vitamin E has shown the ability to inhibit apoptosis. Inhibition of apoptosis by Vitamin E could contribute to its ability to increase the risk of prostate cancer [57]. In contrast, Vitamin E at high doses has also been observed to have prooxidant effects. This could contribute to the observed toxicity of high doses of Vitamin E on specific tissues, specifically the liver [58]. The ability of Vitamin E to exhibit prooxidant effects could lead to the initiation of tumor development [31]. Vitamin E and other antioxidants have the potential to initiate cancer development with prooxidant effects, in addition to protecting cells from oxidative damage and apoptosis, which could lead to the possibility of increased risk for cancer at high doses.

Risk factors

Factors Contributing to Risks With Vitamin E Supplementations

The average patient who consumes Vitamin E daily without exogenous supplementation has a circulating α-tocopherol level of approximately 20 μmol/L. According to current data, consuming Vitamin E-rich foods without additional Vitamin E supplementation has not been proven to produce any adverse effects. In contrast, patients who consume Vitamin E in their diet and intake Vitamin E supplementation have been shown to have approximately 30 μmol/L or higher levels of circulating α-tocopherol. Excessive supplementation of Vitamin E has the potential to cause Vitamin E toxicity. Despite the suggested daily intake of Vitamin E is 15 mg/day, many supplements are produced at much higher daily dosages, ranging from 100-1000 mg per day. Typically, symptoms are not seen in healthy patients until they ingest over 1000 mg of Vitamin E daily [15]. The Food and Nutrition Board has established that doses of up to 1000 mg of Vitamin E per day in adults appear safe, but long-term intake above this recommended amount can lead to an increased risk of adverse health effects [3].

Most clinical trials demonstrating an increased incidence of adverse health events in high-dose Vitamin E supplementation settings involve patients with pre-existing health conditions at baseline. For example, the HOPE and GISSI-Prevenzione trials that link Vitamin E with increased risk of heart failure utilized participants with known underlying health issues. Based on these, it is reasonable to conclude that individuals with a history of heart conditions, diabetes, or vascular disease may be predisposed to experience more detrimental health effects due to taking high doses of Vitamin E [50,59]. Furthermore, Vitamin E's anticoagulant and antiplatelet properties increase the risk of severe bleeding events, such as gastrointestinal bleeds and intracranial hemorrhages, particularly in patients taking warfarin or aspirin, as well as those with inherited platelet or coagulation disorders [60]. Due to the aforementioned risks, Vitamin E supplementation should only be initiated under the guidance of a physician, especially in patients taking prescription medications or with underlying health conditions.

Lifestyle factors, genetic disposition, and drug interactions influence the extent and rate at which different individuals absorb Vitamin E. Numerous studies have demonstrated that smoking, obesity, and alcohol consumption each result in decreased serum α-tocopherol levels [61]. Smoking generates oxidative stress, leading to increased utilization of Vitamin E as an antioxidant to counteract free radical damage; obesity is associated with increased adipose tissue storage of Vitamin E, which reduces its circulating levels; and consumption of alcohol can impair intestinal absorption of Vitamin E and disrupt hepatic transport and metabolism, lowering serum levels [62-64]. Genetic polymorphisms and mutations account for many individual variations in Vitamin E metabolism. For instance, ApoE is a polymorphic protein involved in lipid metabolism with three different isoforms: apoE2, apoE3, and apoE4. In a 2004 study, Lodge et al. determined that subjects with the apoE3 variant had significantly decreased uptake of supplemented alpha-tocopherol compared to subjects with the apoE4 isoform [61]. Any genetic or acquired disorder that disrupts the absorption, transport, or metabolism of lipids will impair the body's ability to absorb Vitamin E from the intestines [61]. For example, patients who suffer from fat malabsorption disorders are more likely to develop deficiencies in Vitamin E. As a result, these patients often need supplementation with water-soluble forms of Vitamin E. Another less common fat malabsorption disorder called abetalipoproteinemia causes severe Vitamin E deficiency, which causes affected individuals to require massive doses of daily supplemental Vitamin E [3].

Supplementing with Vitamin E at doses exceeding 300 mg/day may lead to nutrient-drug interactions with warfarin, aspirin, and cyclosporine A. Various studies involving animals or humans treated with high-dose Vitamin E in combination with either aspirin or warfarin have shown reduced blood clotting and increased bleeding [66]. Steiner (1991) demonstrated that the combination of 325 mg of aspirin and 400 mg of Vitamin E for up to two years was more effective in preventing the recurrence of transient ischemic attacks than in patients taking aspirin alone. This study indicates a potential synergistic effect between Vitamin E and aspirin [33]. Vitamin E is also believed to interact with warfarin due to its possible impact on the metabolism of vitamin K. Corrigan and Ulfers (1981) observed that rats with warfarin-induced vitamin K deficiencies that received injections of Vitamin E in addition to warfarin had reduced activity of mature prothrombin compared to rats treated with warfarin alone. It was concluded that this outcome was likely due to Vitamin E further exacerbating the rats' pre-existing vitamin K deficiency [66]. Vitamin E supplements have also been shown to influence the pharmacokinetics of cyclosporine A, an immunosuppressant used to prevent transplant rejection. Four separate human trials showed that daily doses of greater than 300 mg of Vitamin E in patients taking cyclosporine A resulted in decreased concentrations of cyclosporine A in the participant's blood [33,68-70]. In conclusion, Vitamin E supplementation can reduce the effectiveness of the immunosuppressant cyclosporine A while potentially increasing the effects of antiplatelet and anticoagulants.

Recommendations for Safe Use

There is no recorded evidence of Vitamin E toxicity in individuals who obtain their Vitamin E from food sources alone. Vitamin E deficiency is also extremely rare in humans since a balanced diet is usually sufficient to meet a healthy individual's daily Vitamin E requirements. Vitamin E deficiency is almost exclusive to those with either inherited or acquired fat malabsorption syndromes. In the case of these individuals, Vitamin E supplementation can be beneficial and is often necessary. However, for most healthy patients, it is safest to obtain Vitamin E from dietary sources alone [8].

It has been established that Vitamin E toxicity may occur in healthy patients consuming doses of greater than 1000 mg/day [61]. Furthermore, in patients with pre-existing health conditions or on certain prescription medications, Vitamin E has been shown to have adverse effects in doses as low as 300 mg/day. Patients with baseline health conditions such as heart failure or a history of stroke, as well as patients taking antiplatelet and anticoagulant drugs, should use extreme caution when considering taking Vitamin E supplements. Patients with coagulation or platelet disorders, along with patients taking anticoagulant and antiplatelet medications, should be monitored by their physicians for increased bleeding tendencies while taking Vitamin E [71].

Gaps in Research and Future Directions

The poorly understood effects of Vitamin E on the metabolism of vitamin K is an area that warrants further research. Trials investigating the potential interactions between warfarin and Vitamin E supplementation in humans are limited because of their small sample sizes [72,36]. Furthermore, despite animal trials demonstrating that Vitamin E supplements may worsen vitamin K deficiency induced by warfarin, the mechanism by which it does this remains unknown. One potential explanation is that Vitamin E interferes with vitamin K recycling by inhibiting the activity of vitamin K epoxide reductase (VKOR), reducing the availability of active vitamin K necessary for clotting factor synthesis [73]. Additionally, Vitamin E may compete with vitamin K for hepatic storage and transport, further limiting its bioavailability [60]. Given the uncertainty surrounding these mechanisms, additional research regarding the connection between the metabolism of vitamins E and K would provide a better understanding of the clinical effects Vitamin E supplementation may have on patients taking warfarin [65]. Research on Vitamin E supplementation is also limited due to the lack of clinical trials involving healthy participants. As stated, the results of the HOPE and HOPE-TOO trials raised concern over Vitamin E's potential role in increasing individuals' risk of heart failure [50]. However, it is essential to note that the majority of these studies focus on middle-aged or older adults with existing heart disease or risk factors. Therefore, to better understand if Vitamin E can play a role in preventing coronary heart disease, more extended studies with younger participants taking higher doses of Vitamin E may be necessary.

Additional research can assess whether Vitamin E supplements offer any protective benefits for younger, healthier individuals who do not possess obvious risk factors for adverse cardiovascular events [3]. According to a 2005 meta-analysis, Miller et al. found a statistically significant connection between high-dose Vitamin E supplementation and all-cause mortality. However, since most trials had small sample sizes and only utilized participants with chronic diseases, it is uncertain if these findings can be generalized to healthy adults taking Vitamin E [26]. Overall, further research on healthy participants supplementing with high doses of Vitamin E would help elucidate potential risks associated with Vitamin E supplementation and refine safe dosage recommendations in healthy patients wishing to supplement Vitamin E. However, large-scale trials in healthy individuals have been limited due to ethical concerns regarding potential adverse effects, funding constraints, and the challenge of tracking long-term outcomes in a low-risk population. Addressing these barriers could facilitate future research and provide clearer guidance on Vitamin E supplementation in healthy individuals.

The need for further research is compounded by the equally vital need to increase public awareness of the potential risks associated with high-dosage Vitamin E supplementation. Since supplements such as Vitamin E are easily accessible to the public, individuals may be inclined to utilize them without consulting a healthcare professional and without proper monitoring from their healthcare team. For decades, Vitamin E has been promoted for its benefits as an antioxidant, leading patients to assume that supplementation is entirely safe even in large doses. This raises the concern for potential Vitamin E toxicity in patients who have had this micronutrient marketed to them as beneficial without mentioning the possible risks associated with high doses. In a 2023 narrative review, Li and Wertheimer found that from 2004 to 2021, 79,071 adverse events due to supplement use were reported to the Center for Food Safety and Applied Nutrition. This finding demonstrates the need for more balanced marketing of supplements such as Vitamin E, where the public knows the benefits and the potential risks it may pose to their health [74].

Discussion

Evidence from the various large-scale clinical trials and meta-analyses discussed previously strongly indicates that high-dose supplementation of Vitamin E is associated with increased mortality along with increased risk for cardiovascular events, hemorrhagic stroke, and some cancers. In a 2005 meta-analysis, Miller et al. [26] linked high doses of Vitamin E (>400 IU/day) to an increase in all-cause mortality, potentially through mechanisms such as the 'antioxidant paradox.' This paradox suggests that, instead of offering protection, excessive antioxidant supplementation can paradoxically promote oxidative damage. One proposed explanation is that high levels of exogenous antioxidants like Vitamin E can disrupt the body's redox balance by excessively scavenging ROS. While ROS are often harmful, they also play critical roles in cellular signaling, immune responses, and the regulation of endogenous antioxidant defenses [75]. By indiscriminately neutralizing ROS, high-dose antioxidants may impair essential redox-sensitive signaling pathways, which are needed to activate the body's intrinsic antioxidant defenses [76].

Furthermore, under certain conditions, particularly when Vitamin E is not sufficiently recycled by other antioxidants like vitamin C, it can act as a pro-oxidant rather than an antioxidant. This shift can lead to lipid peroxidation and the formation of secondary free radicals, ultimately exacerbating oxidative stress rather than reducing it [77]. Given the significant impact of oxidative stress on the nervous system [78], this paradoxical effect could be particularly hazardous at the neurological level, potentially contributing to neurodegenerative processes and impairing cellular resilience against oxidative damage.

Regarding Vitamin E's effect on cardiovascular disease, studies such as the HOPE and GISSI-Prevenzione trials demonstrated that Vitamin E can harm patients with pre-existing heart disease [50,51]. In the GISSI-Prevenzione trial, Vitamin E supplementation significantly enhanced the risk of heart failure in cases with poor ventricular function. This effect was likely driven by Vitamin E's potential pro-oxidative properties in certain pathological states, which may have exacerbated myocardial remodeling, impaired cardiac contractility, and interfered with essential signaling pathways involved in heart failure management. Similarly, the HOPE trials demonstrated that Vitamin E supplementation led to a higher incidence of heart failure in patients with pre-existing vascular disease or diabetes mellitus. The findings of these trials suggest that high-dose Vitamin E supplementation may be dangerous to patients with pre-existing heart conditions.

Furthermore, Vitamin E decreased the risk of ischemic stroke by 10% and raised the risk of hemorrhagic stroke by 22% in a 2010 meta-analysis by Schürks et al. [52]. Given that Vitamin E showed only a modest reduction in ischemic stroke risk and such a significant increase in the risk of hemorrhagic stroke, it was determined that the dangers of Vitamin E supplementation in these subjects outweighed the potential benefits [52]. Furthermore, the SELECT trial found that high-dose supplementation of Vitamin E was linked to an increased risk of prostate cancer [31]. The trial involving over 35,000 male subjects concluded that men taking Vitamin E supplements in high doses had a 17% increase in risk of developing prostate cancer due to Vitamin E's potential to inhibit apoptosis, promoting tumor development.

## Conclusions

Overall, the findings in these studies strongly suggest that while low-dose Vitamin E supplementation may be beneficial, high-dose supplementation can pose serious risks to patients, especially those with a history of cardiovascular disease or stroke. Vitamin E is an essential micronutrient involved in reducing oxidative damage and supporting the function of the immune system. Despite its key role in maintaining health, Vitamin E's risks in large doses emphasize the need for a balanced, individualized approach when considering supplementation. Vitamin E supplementation should be utilized cautiously in patients taking anticoagulant and antiplatelet drugs due to its known ability to promote bleeding and deplete vitamin K. Furthermore, patients at risk for developing cancer or those with a history of heart disease or stroke should be wary of Vitamin E supplementation due to its potential to increase their risk for adverse health events. The general population, excluding those with fat malabsorption disorders, can meet their daily requirements of Vitamin E through dietary intake alone. Therefore, most patients should first seek to obtain Vitamin E through a balanced diet, and the decision to initiate supplementation should only be made under the guidance of a physician, carefully weighing the potential risks and benefits.

## References

[1] Jiang Q (2014). Natural forms of vitamin E: metabolism, antioxidant, and anti-inflammatory activities and their role in disease prevention and therapy. Free Radic Biol Med.

[2] Niki E, Noguchi N (2004). Dynamics of antioxidant action of vitamin E. Acc Chem Res.

[3] (2024). Vitamin E. https://ods.od.nih.gov/factsheets/VitaminE-Consumer/.

[4] Australia H (2024). Vitamin E and your health. Healthdirect Australia.

[5] Meydani M (2000). Effect of functional food ingredients: vitamin E modulation of cardiovascular diseases and immune status in the elderly. Am J Clin Nutr.

[6] Tanaydin V, Conings J, Malyar M, van der Hulst R, van der Lei B (2016). The role of topical vitamin E in scar management: a systematic review. Aesthet Surg J.

[7] Aggarwal BB, Sundaram C, Prasad S, Kannappan R (2010). Tocotrienols, the vitamin E of the 21st century: its potential against cancer and other chronic diseases. Biochem Pharmacol.

[8] Rizvi S, Raza ST, Ahmed F, Ahmad A, Abbas S, Mahdi F (2014). The role of vitamin E in human health and some diseases. Sultan Qaboos Univ Med J.

[9] Telford RD (1993). Vitamin E and athletic performance. Asia Pac J Clin Nutr.

[10] Dietrich M, Jacques P, Pencina M (2009). Vitamin E supplement use and the incidence of cardiovascular disease and all-cause mortality in the Framingham Heart Study: does the underlying health status play a role?. Atherosclerosis.

[11] Gaziano JM (2004). Vitamin E and cardiovascular disease: observational studies. Ann N Y Acad Sci.

[12] Shah S, Shiekh Y, Lawrence JA, Ezekwueme F, Alam M, Kunwar S, Gordon DK (2021). A systematic review of effects of vitamin E on the cardiovascular system. Cureus.

[13] Le NK, Kesayan T, Chang JY, Rose DZ (2020). Cryptogenic intracranial hemorrhagic strokes associated with hypervitaminosis E and acutely elevated α-tocopherol levels. J Stroke Cerebrovasc Dis.

[14] Xin J, Jiang X, Ben S (2022). Association between circulating vitamin E and ten common cancers: evidence from large-scale Mendelian randomization analysis and a longitudinal cohort study. BMC Med.

[15] Owen KN, Dewald O (2025). Vitamin E toxicity. StatPearls [Internet].

[16] Sabir B, Hashmi I, Khan MN (2024). Understanding vitamin supplements: misconceptions, risks, and guidelines. World J Pharm Res.

[17] Mehrabadi S, Sadr SS (2020). Administration of vitamin D(3) and E supplements reduces neuronal loss‏ and oxidative stress in a model of rats with Alzheimer's disease. Neurol Res.

[18] Crittenden F, Fang C (2021). Introduction. Yale J Biol Med.

[19] Lobo V, Patil A, Phatak A, Chandra N (2010). Free radicals, antioxidants and functional foods: impact on human health. Pharmacogn Rev.

[20] Halliwell B (2000). The antioxidant paradox. Lancet.

[21] Hampton MB, Orrenius S (2024). Redox regulation of apoptotic cell death. Biofactor.

[22] Roos D, van Bruggen R, Meischl C (2003). Oxidative killing of microbes by neutrophils. Microbes Infect.

[23] Ngo V, Duennwald ML (2022). Nrf2 and oxidative stress: a general overview of mechanisms and implications in human disease. Antioxidants (Basel).

[24] Brugarolas J, Moberg K, Boyd SD, Taya Y, Jacks T, Lees JA (1999). Inhibition of cyclin-dependent kinase 2 by p21 is necessary for retinoblastoma protein-mediated G1 arrest after gamma-irradiation. Proc Natl Acad Sci U S A.

[25] Pearson P, Lewis SA, Britton J, Young IS, Fogarty A (2006). The pro-oxidant activity of high-dose vitamin E supplements in vivo. BioDrugs.

[26] Miller ER, Pastor-Barriuso R, Dalal D, Riemersma RA, Appel LJ, Guallar E (2005). Meta-analysis: high-dosage vitamin E supplementation may increase all-cause mortality. Ann Intern Med.

[27] van Haaften RIM, Haenen GRMM, van Bladeren PJ, Bogaards JJP, Evelo CTA, Bast A (2003). Inhibition of various glutathione S-transferase isoenzymes by RRR-alpha-tocopherol. Toxicol Vitro Int J Publ Assoc BIBRA.

[28] Huang HY, Appel LJ (2003). Supplementation of diets with alpha-tocopherol reduces serum concentrations of gamma- and delta-tocopherol in humans. J Nutr.

[29] Bowry VW, Stocker R (2024). Tocopherol-mediated peroxidation. The prooxidant effect of Vitamin E on the radical-initiated oxidation of human low-density lipoprotein. J Am Chem Soc.

[30] Vivarelli F, Canistro D, Cirillo S (2019). Co-carcinogenic effects of vitamin E in prostate. Sci Rep.

[31] Klein EA, Thompson IM Jr, Tangen CM (2011). Vitamin E and the risk of prostate cancer: the Selenium and Vitamin E Cancer Prevention Trial (SELECT). JAMA.

[32] Njoroge RN, Unno K, Zhao JC (2017). Organoids model distinct Vitamin E effects at different stages of prostate cancer evolution. Sci Rep.

[33] Steiner M (1991). Influence of vitamin E on platelet function in humans. J Am Coll Nutr.

[34] Freedman JE, Farhat JH, Loscalzo J, Keaney JF Jr (1996). α-Tocopherol inhibits aggregation of human platelets by a protein kinase C-dependent mechanism. Circulation.

[35] Dowd P, Zheng ZB (1995). On the mechanism of the anticlotting action of vitamin E quinone. Proc Natl Acad Sci U S A.

[36] Pastori D, Carnevale R, Cangemi R (2013). Vitamin E serum levels and bleeding risk in patients receiving oral anticoagulant therapy: a retrospective cohort study. J Am Heart Assoc.

[37] Loh HC, Lim R, Lee KW (2021). Effects of vitamin E on stroke: a systematic review with meta-analysis and trial sequential analysis. Stroke Vasc Neurol.

[38] Tripathi S, Nath M, Misra S, Kumar P (2024). From A to E: uniting vitamins against stroke risk—a systematic review and network meta-analysis. Eur J Clin Invest.

[39] Medina J, Gupta V (2023). Vitamin E. StatPearls [Internet].

[40] Chin KY, Ima-Nirwana S (2014). The effects of α-tocopherol on bone: a double-edged sword?. Nutrients.

[41] (2024). Is Vitamin E bad for your bones?. Harv Health Lett.

[42] Abrol R, Kaushik R, Goel D, Sama S, Kaushik RM, Kala M (2023). Vitamin E-induced coagulopathy in a young patient: a case report. J Med Case Rep.

[43] Asbaghi O, Sadeghian M, Nazarian B (2020). The effect of vitamin E supplementation on selected inflammatory biomarkers in adults: a systematic review and meta-analysis of randomized clinical trials. Sci Rep.

[44] Lewis ED, Meydani SN, Wu D (2019). Regulatory role of vitamin E in the immune system and inflammation. IUBMB Life.

[45] Prasad JS (1980). Effect of vitamin E supplementation on leukocyte function. Am J Clin Nutr.

[46] Baehner RL, Boxer LA, Allen JM, Davis J (1977). Autooxidation as a basis for altered function by polymorphonuclear leukocytes. Blood.

[47] European Food Safety Authority (EFSA), de Sesmaisons Lecarré A, Fabiani L, de Sousa RF, Horvath Z (2022). Protocol for the intake assessments performed in the context of the revision of Tolerable Upper Intake Levels for selected nutrients. EFSA Support Publ.

[48] Turck D, Bohn T, Castenmiller J (2023). Scientific opinion on the tolerable upper intake level for vitamin D, including the derivation of a conversion factor for calcidiol monohydrate. EFSA J.

[49] Ford ES, Ajani UA, Mokdad AH (2005). Brief communication: the prevalence of high intake of vitamin E from the use of supplements among U.S. adults. Ann Intern Med.

[50] Lonn E, Bosch J, Yusuf S (2005). Effects of long-term vitamin E supplementation on cardiovascular events and cancer: a randomized controlled trial. JAMA.

[51] Marchioli R, Levantesi G, Macchia A (2006). Vitamin E increases the risk of developing heart failure after myocardial infarction: results from the GISSI-Prevenzione trial. J Cardiovasc Med (Hagerstown).

[52] Schürks M, Glynn RJ, Rist PM, Tzourio C, Kurth T (2010). Effects of vitamin E on stroke subtypes: meta-analysis of randomised controlled trials. BMJ.

[53] Traber MG (2008). Vitamin E and K interactions - a 50-year-old problem. Nutr Rev.

[54] Furie B, Bouchard BA, Furie BC (1999). Vitamin K-dependent biosynthesis of gamma-carboxyglutamic acid. Blood.

[55] Ferro D, Basili S, Praticó D, Iuliano L, FitzGerald GA, Violi F (1999). Vitamin E reduces monocyte tissue factor expression in cirrhotic patients. Blood.

[56] Qin F, Yan C, Patel R, Liu W, Dong E (2006). Vitamins C and E attenuate apoptosis, beta-adrenergic receptor desensitization, and sarcoplasmic reticular Ca2+ ATPase downregulation after myocardial infarction. Free Radic Biol Med.

[57] Tafazoli S, Wright JS, O'Brien PJ (2005). Prooxidant and antioxidant activity of vitamin E analogues and troglitazone. Chem Res Toxicol.

[58] McCall MR, Frei B (1999). Can antioxidant vitamins materially reduce oxidative damage in humans?. Free Radic Biol Med.

[59] GISSI-Prevenzione Investigators (1999). Dietary supplementation with n-3 polyunsaturated fatty acids and Vitamin E after myocardial infarction: results of the GISSI-Prevenzione trial. Lancet Lond Engl.

[60] Schmölz L, Birringer M, Lorkowski S, Wallert M (2016). Complexity of vitamin E metabolism. World J Biol Chem.

[61] Lodge JK, Hall WL, Jeanes YM, Proteggente AR (2004). Physiological factors influencing vitamin E biokinetics. Ann N Y Acad Sci.

[62] Bruno RS, Traber MG (2005). Cigarette smoke alters human vitamin E requirements. J Nutr.

[63] Alvarado-Ramos K, Bravo-Núñez Á, Vairo D, Sabran C, Landrier JF, Reboul E (2024). Overweight leads to an increase in vitamin E absorption and status in mice. Mol Nutr Food Res.

[64] Butts M, Sundaram VL, Murughiyan U, Borthakur A, Singh S (2023). The influence of alcohol consumption on intestinal nutrient absorption: a comprehensive review. Nutrients.

[65] Podszun M, Frank J (2014). Vitamin E-drug interactions: molecular basis and clinical relevance. Nutr Res Rev.

[66] Corrigan JJ Jr, Ulfers LL (1981). Effect of vitamin E on prothrombin levels in warfarin-induced vitamin K deficiency. Am J Clin Nutr.

[67] Bárány P, Stenvinkel P, Ottosson-Seeberger A, Alvestrand A, Morrow J, Roberts JJ 2nd, Salahudeen AK (2001). Effect of 6 weeks of vitamin E administration on renal haemodynamic alterations following a single dose of neoral in healthy volunteers. Nephrol Dial Transplant.

[68] Lake KD, Aaronson KD, Gorman LE, Pagani FD, Koelling TM (2005). Effect of oral vitamin E and C therapy on calcineurin inhibitor levels in heart transplant recipients. J Heart Lung Transplant.

[69] Blackhall ML, Fassett RG, Sharman JE, Geraghty DP, Coombes JS (2005). Effects of antioxidant supplementation on blood cyclosporin A and glomerular filtration rate in renal transplant recipients. Nephrol Dial Transplant.

[70] de Vries AP, Oterdoom LH, Gans RO, Bakker SJ (2006). Supplementation with anti-oxidants Vitamin C and E decreases cyclosporine A trough-levels in renal transplant recipients. Nephrol Dial Transplant.

[71] Keen MA, Hassan I (2016). Vitamin E in dermatology. Indian Dermatol Online J.

[72] Kim JM, White RH (19961). Effect of vitamin E on the anticoagulant response to warfarin. Am J Cardiol.

[73] Wu S, Chen X, Jin DY, Stafford DW, Pedersen LG, Tie JK (2018). Warfarin and vitamin K epoxide reductase: a molecular accounting for observed inhibition. Blood.

[74] Li W, Wertheimer A (2023). Narrative review: the FDA's perfunctory approach of dietary supplement regulations giving rise to copious reports of adverse events. Innov Pharm.

[75] Bouayed J, Bohn T (2010). Exogenous antioxidants—Double-edged swords in cellular redox state: health beneficial effects at physiologic doses versus deleterious effects at high doses. Oxid Med Cell Longev.

[76] Rahal A, Kumar A, Singh V, Yadav B, Tiwari R, Chakraborty S, Dhama K (2014). Oxidative stress, prooxidants, and antioxidants: the interplay. Biomed Res Int.

[77] Miyazawa T, Burdeos GC, Itaya M, Nakagawa K, Miyazawa T (2019). Vitamin E: regulatory redox interactions. IUBMB Life.

[78] Rekatsina M, Paladini A, Piroli A, Zis P, Pergolizzi JV, Varrassi G (2020). Pathophysiology and therapeutic perspectives of oxidative stress and neurodegenerative diseases: a narrative review. Adv Ther.

